# General Therapeutic Significance of Collargol

**Published:** 1910-04

**Authors:** 


### ABSTRACTS

---

### General Therapeutic Significance of Collargol

DR. H. HUCHARD, Chief Physician of the Hôspital Necker in Paris, editor of the *Journal des Praticiens* and Dr. Ch. Fiessinger, correspondent member of the Académie de Médecine, have published in the above journal a series of articles under the joint title of "La thérapeutique en vingt médicaments" (Therapeutics in twenty medicaments.) One separate article is devoted to each one of the following therapeutic agents: sodium salicylate, quinine, mercury, iodide of potassium, iron, specific sera, collargol and the metallic ferments, opotherapeutic medicaments, subnitrate of bismuth, theobromine purgatives, digitalis, bicarbonate of soda, arsenic, opium, belladonna, bromide of potassium, nitrites, ergot and antipyrine.

In the article on "Collargol and the metallic ferments," which appeared in No. 36 (September 4, 1909), the authors give a brief introductory consideration to the physical, chemical and therapeutic properties of colloids in general, set forth the essential differences between collargol and the metallic ferments and proceed as follows:

Credé (of Dresden, Germany) introduced collargol into therapeutics and Netter was its sponsor in France.[1] The general enthusiasm, with which it was received, is still in everybody's memory. Now, after, several years of experience with the remedy, the over-enthusiastic have found it necessary to modify some of their earlier opinions, but collargol has kept its distinct place in the therapy of certain infectious diseases. It is a remedial agent, which principally favors the process of defence by causing an abundant leukocytosis.[2] Until each disease will have its specific serum, which now seems to have been realized in cerebral meningitis and septicemia, collargol will retain a place of honor in therapy.

Collargol may be employed in form of a 15 per cent. ointment (unguentum Credé), per os, and by intravenous, intramuscular, intrarachidian, intrapleural, intravesical, intrarenal, vaginal, uterine or urethral injection.

The ointment is preferably applied at the elbow-joint, the axilla, buttock or the groin, the site of inunction being previously prepared by thorough brushing with soap and subsequent washing off with ether. The ointment is vigorously rubbed in for fifteen to twenty minutes and the site covered with cotton and oil silk, without previously removing the non-absorbed portion.

After six to twelve hours, the site is cleansed and prepared again and the operation repeated two or three times every twenty-four hours, using 45 grains of ointment for each inunction. A metallic taste in the patient's mouth will indicate successful absorption, and, together with the subsidence of the subjective symptoms, serves as a criterion when to interrupt the treatment.

Intravenous injections are best made at the elbow joint with a syringe holding about 20 c.c., needle and barrel of which have been carefully sterilised. The site is thoroughly brushed with soap and washed with alcohol and ether. An elastic ligature is then placed around the arm, causing the vein to rise, the needle is introduced slantingly, a little blood drawn (to assure it being well inside the vein), the syringe attached and 5 to 15 c.c. (1¼ to 3¾ fl. drams) of a 1 to 2½ per cent. solution (according to the gravity of the case) slowly injected. In the majority of cases the organism reacts by a chill, followed by a transitory rise in temperature and muscular contractions, all of which phenomena pass off soon and are precursory to a fall of temperature with improvement of the general condition.[3]

For the administration of intramuscular injections the authors prefer to use an isotonic colloidal silver preparation, of which they inject 5 to 10 c.c. (1¼ to 2½ fl. drams) twice a day, once a day, or every other day, according to the individual requirements of the case.

Intrarachidian injections of collargol, first used in the treatment of meningitis, have been replaced, at least in epidemic cerebral meningitis, by injections of the specific serum. However, excellent results with collargol in chronic meningitis and myelitis have been reported. Generally speaking, intrarachidian injections of this colloidal silver preparation are more effective than intravenous administrations in cerebral, medullar and meningeal infections. Mosny and Pinard[4] have reported a recovery from spasmodic syphilitic paraplegia by intrarachidian injections of collargol, and Larens[5] has demonstrated that, administered in this manner, it does not pass through the meningeal and submeningeal spaces.

Intrapleural injections of collargol have rendered valuable services in the treatment of pleural suppurations. Daily injections of 20 to 30 c.c. (5 fl. drams to 1 fl. oz.) of a 1 per cent. collargol solution into the pleural cavity advantageously replace the injections with Van Swieten's solution or naphtol-camphor. In a case of purulent pleurisy and septico-pyemia, following typhoid fever, Triboulet and his collaborators[6] have had signal success with this treatment.

Intravesical injections with collargol solutions on a sliding scale (1, 2 and 3 per cent. in strength) have been recommended by Jeanbran[7] and are now generally known to produce the same effect as installations of silver nitrate, but are not painful. Pasteau[8] prefers to let the collargol solution remain in the bladder for some time. He also employs intrarenal injections of 1 to 4 per cent. solutions, by means of the catheter, preceded by an ordinary antiseptic lavage. The authors have used this method frequently with the best of success in pyelo-nephritis and report one case in particular, in which the high temperature, which had prevailed uninterruptedly for three weeks, disappeared under this treatment in three days and complete recovery ensued promptly. They recommend that, in withdrawing the catheter, portions of the collargol solution be deposited all along the genitourinary tract.

Urethral injections can be practised in general urinary infections to good advantage, but not to the exclusion of other therapeutic measures (internal, surgical, and the like). Owing to the large quantities required and the consequent high cost, vaginal injections with collargol are not frequently employed. In blennorrhagia, installations of ½ per cent. solutions are recommended,[9] and in puerperal septicemia, as an adjunct to the other forms of collargol therapy, (see further on), uterine tamponage with the ointment or solution render excellent services.

Per os, the authors frequently administer collargol in pills of ¾ grains each, daily dose four pills, or flavored solution, containing 3 grains in 6 ounces of distilled water, of which a soup-spoonful is given every three hours. Netter[10] recommends 7½ grains in 3 ounces of distilled water, with flavors, of which two to four dessert-spoonfuls are to be given in the course of twenty-four hours. Loebe[11] employs collargol solutions for stomach lavages with success.

The remaining diseases, in the treatment of which collargol has found employment, are divided by the authors into two groups: Those, in which their own experience and that of others fully convinced them of its therapeutic efficiency, and those, in which they consider its action doubtful, although sporadic successes have been reported.

In phlegmons and local inflammations, collargol, both by inunction and by intravenous injection, has time and time again rendered the best of services to the authors. Ribadeau-Dumas, Bailleul[12] and more recently, Delbet,[13] who has used it in suppurative arthritis, osteomyelitis, post-operative septicemia and otitis, have reported favorably. Even when surgical intervention becomes necessary, collargol should be used prophylactically. Heroic doses are often necessary and may be given without hesitation. Intravenous injections of 1½ grains may be given and repeated every two hours, if necessary. Brocq successfully treats malignant syphilides with collargol ointment. In tuberculous ganglia Breton,[14] after rigorous asepsis, aspirates the pus and injects 1 to 20 c.c. (15 minims to 5 fl. drams) of collargol solution, until the tension is felt by the patient. After a few instants, part of the liquid is withdrawn and the site of injection closed with collodium. This procedure is repeated every four or five days. It is in puerperal septicemia, however, that the most brilliant results with collargol have been achieved. The authors in accord with Breton, Rouet,[15] Hermoglu,[16] Bonnaire and Jeannin,[17] recommend intravenous injections of 10 to 15 c.c. (2½ to 3¾ fl. drams) of 1 per cent. collargol solution every day, or on alternate days, according to the gravity of the case, supported if possible, by inunctions and uterine tamponage with collargol ointment. They claim favorable results in 76 per cent. of the cases treated. The prognosis is unfavorable, if the temperature rises again after having once remained low for several hours. In septic endocarditis collargol must be used in the very earliest stages to be effective, and under those conditions, is unrivaled in the treatment of this grave affection. Netter, Chauffard and others have reported positive results. Daily intravenous injections of 5 to 10 c.c. (1¼ to 2½ fl. drams) of a 1 per cent. solution are given, until improvement is noticed. After that, inunctions with collargol ointment or intramuscular injections of isotonic colloidal silver should be employed for a short time. Among the gastrointestinal infections paratyphoid fever, above all, has been successfully treated with collargol. This disease, usually the sequel of alimentary intoxications, and caused by the bacillus of Gaertner, fluctuates in its manifestations from ordinary gastro-febrile symptoms to those of the gravest typhoid fever. Netter and Ribadeau-Dumas describe an intermittent form of it, which yields best to collargol and hydrotherapy. The authors suggest, that in absence of a known typhoid epidemic, physicians begin by administering collargol per os or by inunction, until the Widal test has been made. Netter has also successfully used collargol per os (1½ to 3 grains) in tubercular enteritis.

The action of collargol is considered doubtful by the authors in mixed diphtheritic infections, in which Netter[18] has suggested the employment of collargol either by inunction, or intravenous injection, in combination with large doses of antidiptheritic serum. It is manifestly impossible under these conditions to prove, how much of the credit is due to collargol and how much to the serum. Favorable results with collargol have also been reported in the treatment of grippe, typhoid fever, acute tuberculosis and broncho-pneumonia, but the authors have not been able to confirm them by their own experience. They have also been unsuccessful with it in phlebitis. Opportunity has so far not presented itself to the authors to try collargol in the treatment of epilepsy, in which it has been recommended by Netter, who claims that under its influence smaller doses of bromide can be used with equally good results. In ocular inflammations (conjunctivitis with hypopyon) Troussean[19] reports good results by the use of collargol ointment. The authors have at times had fair results, without being entirely convinced of its efficiency in this field. In articular rheumatism they prefer to first exhaust all the possibilities of salicylate of soda. In certain subacute cases, where this drug has failed, intravenous injections of 4 to 8 c.c. (1 to 2 fl. drams) of a 2 per cent. solution of collargol,[20] seem to cause a fall of temperature and to allay the pain; the remedy should be given a trial under such circumstances. De

Cisternes[21] recommended inunctions with collargol ointment in tubercular rheumatism, but other observers, including the authors, have not been able to confirm his results.

Dr. H. Albrecht, First Assistant to Professor J. A. Amann at the Second Gynecological Clinic, Munich, Germany, in a paper read before the Munich Gynecological Society on June 17, and published in the *Muenchener Medizinische Wochenschrift,* No. 51, December 21, 1909, critically reviews the therapeutic significance of collargolum at the hands of forty-five cases, which have been treated at the clinic in the past eighteen months and are representative of a great variety of septic infections. Of the four modes of administration—by inunction, per rectum, intravenously and per os—the author recommends only the gradual intravenous injection of 1 to 2 c.c. of 5-10 per cent. suspensions. He considers collargolum a valuable therapeutic agent in cases of septicemia and pyemia of medium gravity, in severe resorptive fevers, accompanied by obstinate and enduring toxanemia, more particularly in all such cases, where in spite of local treatment and apparent localisation of the infective process, unchanged high temperature and pulse indicate the presence of a deep-going tissue involvement and a progressive surcharge of the blood with toxins. In these cases the reaction is so prompt and distinctive, that it is impossible to underrate the efficiency of collargolum. According to the author, collargolum should be given a trial in all cases of puerperal infection, because it is impossible to diagnose the gravity of the infection at the beginning, and also on account of the prompt reduction of temperature and improvement of the general condition which it brings about. In very severe bacteriemiæ, in purulent peritonitis, parametritis exsudativa and in virulent localised suppurations, collargol did not prove of value. The author emphasises the usefulness of local admin-

1. Bull. Soc. Méd. Hôpit., December 12, 1902 and January 16, 1903.
2. Schreiber, Obstétrique, Paris, May 21, 1908.
3. A. Guérin, Journ. des Practiciens, 1908. p. 604.
4. Bull. Soc. Méd Hôpit., June 15, 1908.
5. Ibid., November 21, 1907.
6. Ibid., June 26, 1907.
7. Bull. Assoc. Franç. d'Urologie, August 12, 1907.
8. Paris chirurgical. April, 1909, p. 301.
9. Tansard, Journ. des Praticiens. 1905, No. 20.
10. Bull. Soc. Méd. Hôpit., April 22, 1904.
11. Therapie d Gegenwart, 1904.
12. Journ des Praticiens, 1905, p. 231.
13. Paris chirurgical, April, 1909.
14. Journ. des Praticiens, 1905, p. 482.
15. Ibid, 1907, p. 581.
16. Ibid., 1907, p. 552.
17. Obstétrique, 1908, No. 2.
18. Th. Baudoin, La diphtérie à l'hôpital Trousseau, S. Steinheil, 1909.
19. Journ. des Praticiens, 1903, p. 196.
20. R. Georg, Muench. Medizin. Wochenschr., 1906, April 17.
21. Journ des Praticiens, 1904, No. 34.

istrations of collargolum in acute cystitis and in pyelitis, as well as the now unquestionably proved innocuousness of the product. He does not ascribe antibacterial action to collargolum and questions its leukocytogenetic properties but believes that its efficiency is due to a catalytic action, consisting of ready absorption, accelerated oxydation and consequent decreased virulency of the toxins.

Professor Doederlein, *Proc. of the Munich Gynecol. Soc.*, June 17, 1909, in the discussion of the above paper stated that collargolum therapy is unquestionably frequently of value and warned against the therapeutic nihilism which actuated some of the exaggeratedly adverse opinions.

Dr. A. Abrams in his textbook on "Diagnostic Therapeutics" (New York, 1910, Rebman Co.) refers to collargol in the following manner: Collargol is an allotropic form of silver, which is soluble in water and the secretions of the body. It is chiefly used externally as a salve (unguentum Credé) and has a remarkable influence in many infectious diseases. In many instances it may be necessary in the diagnosis to determine the part played by the infection in a given symptomatic picture, and for this purpose collargol is indicated. The efficiency of this, like many other drugs, is accentuated by intravenous injection. By the latter route, the dose varies from ½ to 5-6 of a grain. (Here follows a description of the technic of intravenous injections.) Collargol, if effective in septicemia, causes the temperature to fall (within 6 hours), with improvement of the subjective symptoms.

IN DISEASES OF THE PUERPERIUM.

Dr. G. Thomson, Red Cross Hospital, Odessa, Russia; *Therap. Obosrenije,* 1909, Vol. 2, No. 2, discusses "Collargol treatment of septic conditions post partum vel abortum." In the hospital named, collargol is used intravenously in all septic conditions where rapid action is desired. Per rectum the remedy is employed in all lighter forms of sepsis, in local affections and in general conditions at the very beginning of the invasion, and thus used, the results in many cases have been very gratifying. The author won the impression that early use of the remedy greatly lessened the gravity and course of the affection. Also from collargol inunction (unguentum Credé) sudden improvement was frequently noted. Sometimes, shortly after the injection, there develops a chill, followed by rise in temperature—reactions of the organism. Ultimately, the remedy causes in the many favorable cases, which have come to the author's attention, a lowering of the temperature, slowing of the pulse and improvement in the general, as well as the subjective symptoms. Collargol is not a specific in septic infection, but has been found extremely useful in that condition.

Dr. Barton Cooke Hirst, Professor of Obstetrics, Univ. of Pennsylvania, in his *Textbook of Obstetrics.* (Sixth edition. W. B. Saunders, Philadelphia and London), states in the chapter on "puerperal septicemia": colloidal silver (collargol) by inunction (Credé's ointment), by intravenous injection (3 to 5 c.c. of a 2 per cent. solution of collargol, repeated three to four times), and by rectal injection, has its advocates. It has secured extensive clinical trials in Chrobak's Clinic, in the Charité of Berlin, in Budapest and in other large maternities. The verdict, on the whole, is favorable. As an adjunct to other treatment it may be recommended.

